# Vesical Calculi and Female Pelvic Organ Prolapse: A Case Report and Literature Review

**DOI:** 10.7759/cureus.44578

**Published:** 2023-09-02

**Authors:** Saeed AlSary, Eman F Al-Zahrani, Maha Al Baalharith

**Affiliations:** 1 Urogynecology and Reconstructive Pelvic Surgery, King Abdul-Aziz Medical City, Ministry of National Guard Health Affairs, Riyadh, SAU; 2 Obstetrics and Gynecology, King Abdullah International Medical Research Center, Riyadh, SAU

**Keywords:** uterovaginal prolapse, urolithiasis, bladder stones, pelvic organ prolapse, vesical calculi

## Abstract

This study presents a case report and reviews the literature on the simultaneous occurrence of advanced uterovaginal prolapse and urolithiasis, aiming to provide a comprehensive analysis of the reported cases. A thorough search was conducted in PubMed and Google Scholar; the search strategy included specific keywords and terms related to both conditions aiming to identify relevant case reports describing the association between advanced uterovaginal prolapse and urolithiasis; a total of 22 case reports were found in English literature.

We present a case report of a 56-year-old woman, para 4, presenting with complaints of vaginal bulge and urinary symptoms. Upon examination, a complete procidentia with superficial ulceration was observed. During the reduction of the uterus, multiple small stones were noted coming through the urethral meatus. The patient underwent a vaginal hysterectomy, and the bladder stone was successfully extracted through vaginal cystotomy without complications. Our case report highlights the association between vesical calculi and female high-grade pelvic organ prolapse. Managing bladder stones in the context of pelvic organ prolapse can be challenging and vary significantly, reflecting the individual patient characteristics and surgeon preferences. The lack of standardized guidelines for managing bladder stones in the presence of pelvic organ prolapse highlights the need for further research.

## Introduction

Vesical calculi and female pelvic organ prolapse (POP) are distinct yet linked conditions that significantly impact women's quality of life [1]. Vesical calculi, commonly known as bladder stones, are calcified mineral buildup that forms within the bladder [2]. POP refers to descending pelvic organs, such as the bladder, uterus, or rectum, into or outside the vaginal canal [3]. Although they are discrete entities, there is evidence suggesting a potential link between these two conditions which warrants further investigation [4].

There are 22 case reports in the English literature that describe bladder calculi as a sequelae of advanced POP. These reports highlight the correlation between the two conditions, emphasizing the potential role of POP in the development of bladder stones. In addition to reviewing literature and case reports, an in-depth analysis of the relationship between vesical calculi and female POP is still needed.

We present a case study of a 56-year-old woman with incarcerated procidentia, a severe form of POP, who underwent a vaginal hysterectomy to treat advanced POP. A large bladder stone was incidentally discovered intraoperatively, thus highlighting the importance of realizing how these two conditions relate. We aim to analyze the existing evidence, and identify the likely associations, shared risk factors, and underlying mechanisms between vesical calculi and female POP.

Methodology

A systematic search of multiple databases was conducted to identify relevant data for this literature review on the simultaneous occurrence of advanced uterovaginal prolapse with urolithiasis. The primary databases used were PubMed and Google Scholar.

An extensive search strategy was developed to retrieve all relevant articles and case reports up to the date of this publication and minimize the risk of missing any important information. The search terms and keywords included uterovaginal prolapse, urolithiasis, bladder stones, POP, and their synonyms and variations. A total of 27 cases were initially identified. However, five cases were excluded due to being written in other non-English languages, including French, Italian, Bulgarian, and German. Subsequently, bringing the total number of cases included in the study to 22 cases.

It is important to emphasize that the patient reported in this paper provided informed consent for publishing her case and accompanying pictures, adhering to the CARE Guidelines (Case Report Guidelines) and ensuring patient confidentiality and compliance with ethical considerations.

## Case presentation

Our case involves a 56-year-old woman who presented to our clinic in 2021 with complaints of increasing vaginal bulge that she had been experiencing since 2011. She also reported urinary symptoms, including leakage with effort, urgency with urge incontinence, and occasional painful urination. The patient had a medical history of hypothyroidism and cholelithiasis.

**Figure 1:**
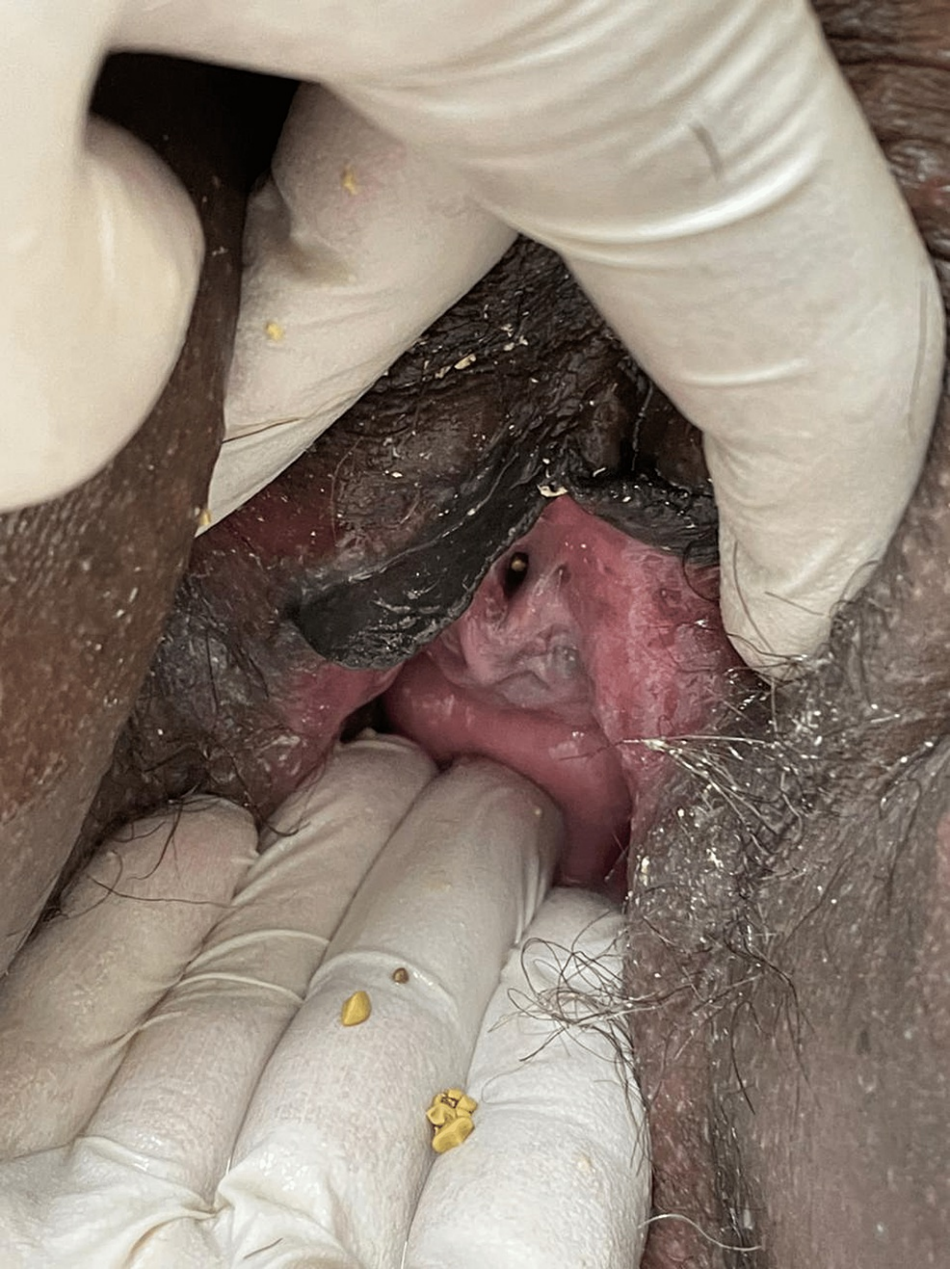
Small bladder stones expressed through the urethral meatus after manual reduction of uterine prolapse.

Upon examination, a complete uterovaginal prolapse was observed, measuring approximately 15 x 15 cm, with superficial ulceration and tissue sloughing upon touch. The uterus was reduced with difficulty, and a size 8 (gel horn) pessary was fitted. During the reduction of the uterus, multiple small stones were noted coming through the urethral meatus (Figure 1). A clean catch urine sample was obtained after an in/out Foley catheter was inserted, and the sample was sent for culture and analysis.

The patient was counseled and agreed to undergo a vaginal hysterectomy with sacrospinous fixation. However, until the scheduled operation, she was put on daily application of conjugated estrogen vaginal cream for three weeks, along with the vaginal pessary, to reduce the swelling of the vaginal wall and promote healing of the ulcers. Renal ultrasound and cystogram were arranged for further evaluation.

**Figure 2:**
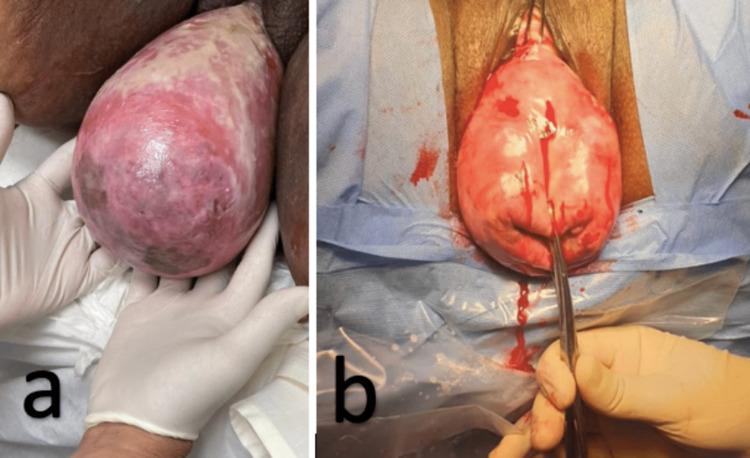
(a) Showing the prolapsed uterus with swelling, ulcers, and sloughed tissue. (b) Intra-op image of healed vaginal tissue after estrogen cream along with gel horn pessary.

The pessary was in place three weeks after her initial visit, and the cervix and vaginal tissue appeared to be healed (Figure 2). Laboratory results and imaging were reviewed, revealing normal renal function and a negative urine culture for infection. The renal ultrasound showed no renal stones or hydronephrosis, but the urinary bladder appears partially distended and filled with small stones.

**Figure 3:**
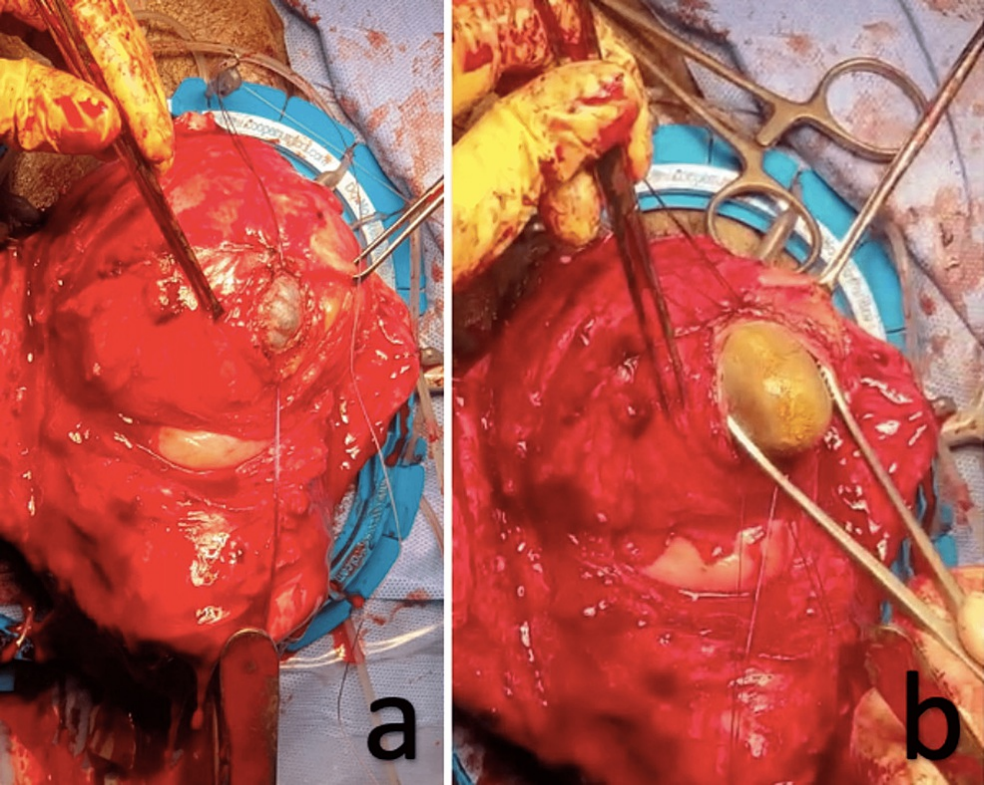
(a, b) Transvaginal cystotomy and bladder stone removal.

The intraoperative course began with a diagnostic cystoscopy, confirming the presence and patency of ureteric orifices and normal bladder anatomy. A single bladder stone, measuring approximately 4 x 4 cm, was observed, and no more small stones. Taking this finding into account, we proceeded with a vaginal hysterectomy as planned. After the removal of the uterus and before the closure of the vault, the bladder was instilled with approximately 200 cc of normal saline to put it under tension. A 3 cm incision was made layer by layer through the dome of the bladder (Figure 3).

**Figure 4:**
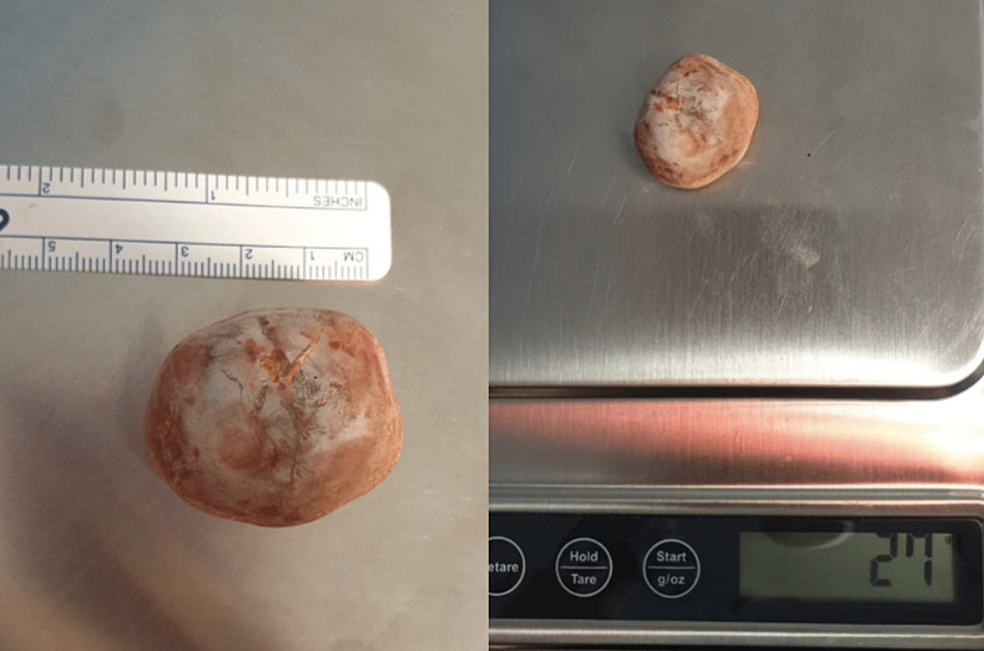
Extracted bladder stone

Moreover, the large stone measuring around 4 cm and weighing 27 grams (Figure 4) was successfully extracted vaginally without complications. The bladder was then closed in two layers, and the integrity of the cystotomy line was checked using methylene blue, confirming a watertight closure. Subsequently, anterior vaginal wall repair, posterior vaginal wall repair, and sacrospinous fixation were performed without issues.

The patient's postoperative course was uneventful, and she was discharged on the second-day post-op in stable condition on a Foley catheter. CT cystogram performed on the 14th day post-op showed an intact bladder, leading to the removal of the Foley catheter. The patient was followed up in the clinic twice after the procedure, and until the date of this publication, she is doing well with no urinary or POP symptoms. This case highlights the importance of identifying and managing the concurrence of vesical calculi and female POP, contributing to the growing body of literature on this association.

## Discussion

Literature review

We analyzed relevant case reports involving patients with similar presentations and complications. Table 1 summarizes the key findings from these case reports.

**Table 1:** Key findings from different case reports POP = pelvic organ prolapse, N/R = not reported, BSO = bilateral salpingo-oophorectomy, US = ultrasound, KUB = kidney, ureter, bladder, IVP = intravenous pyelogram, LUTS = lower urinary tract symptoms, SCLP = sacrocolpopexy, PR = posterior vaginal wall repair, AR = anterior vaginal wall repair, SUI = stress urinary incontinence, UUI = urge urinary incontinence, APR = anterior and posterior vaginal wall repair, SSLF = sacrospinous ligament fixation

Study	Urinary Symptoms	Grade of POP	Duration in POP	Imaging Done	POP Surgery	Vesicle Stone Surgery Route	No. of Stones	Largest Stone	Weight of Stone
Chaudhary et al., 2022 [5]	Suprapubic pain, difficulty passing urine, incontinence with nocturnal enuresis	Procidentia, rectal prolapse	N/R	MRI pelvis	Subtotal hysterectomy, SCLP, burch, rectosigmoidectomy with anastomosis, PR	Transabdominal cystolithotomy	N/R	N/R	N/R
Yuan & Ng, 2022 [6]	SUI, UUI	Procidentia with a gritty sensation	4 years	Pelvic US KUB X-ray KUB US	Prolapse reduction with pessary, interval vaginal hysterectomy, Mcall culdoplasty, APR	Spontaneous expulsion before surgery	39	Ranging from >10 mm to <10 mm	140 g
Garg et al., 2019 [7]	LUTS, dysuria	Grade 3 cystocele	10 years	KUB Xray Abdominal US	Vaginal hysterectomy, AR	Transabdominal cystolithotomy	Multiple	N/R	N/R
Herimath et al., 2019 [8]	Acute urinary retention, dysuria, abdominal pain	Vault prolapse	10 years	KUB X-ray CT KUB Renal/Pelvis US	Laparoscopic SCLP	Transurethral cystolitholapaxy with stone removal system	2	N/R	N/R
Thompson et al., 2018 [1]	Difficulty passing urine	Procidentia, rectal prolapse with intussusception	7 yrs	CT scan	Abdominal subtotal hysterectomy, rectosigmoid colectomy, SCLP, burch, PR, perineorrhaphy	Transabdominal cystolithotomy	Multiple	5 cm	191 g
Saha et al., 2016 [9]	Difficulty passing urine	Grade 3 uterovaginal prolapse	20 years	KUB US Pelvic US Plain X-Ray pelvis and Abdomen	Vaginal hysterectomy, APR	Transabdominal cystolithotomy	N/R	6 cm x 8 cm	N/R
Chhabra & Raman, 2015 [10]	Difficulty passing urine, dysuria	Procidentia	5-7 years	Abdominopelvic US IVP	Vaginal hysterectomy, APR	Transabdominal cystolithotomy	N/R	N/R	N/R
	Difficulty passing urine	Procidentia	5 years	Cystoscopy Abdominopelvic US IVP	Vaginal hysterectomy, APR	Transabdominal cystolithotomy	N/R	N/R	N/R
	Difficulty passing urine	Grade 3 uterovaginal prolapse	1 year	Abdominopelvic US IVP	Vaginal hysterectomy, vaginectomy, APR, pelvic lymphadenectomy	Transabdominal cystolithotomy	N/R	N/R	N/R
Hudson et al., 2014 [11]	Occasional hematuria, involuntary urinary leakage	Grade 4 uterine prolapse, enterocele	22 years	CT pelvis	Lefort colpocleisis without hysterectomy, perineorrhaphy	Transvaginal cystolithotomy	11	2.5 cm	45 g
Rajamaheswari et al., 2012 [12]	Frequency, dysuria, UUI	Irreducible Grade 4 uterine prolapse	20 years	Pelvic US Renal US Pelvis X-ray IVP CT Pelvis	Exploratory laparotomy, vaginal hysterectomy	Transabdominal cystolithotomy	Multiple	10 cm x 8 cm	N/R
Naidu et al., 2011 [4]	Acute urinary retention, frequency, weak urine stream	Grade 4 cystocele+rectocele, procidentia with inverted uterus	N/R	Pelvic US	Vaginal hysterectomy, colpocleisis	Transvaginal cystolithotomy	30	2.5 x 3.0 cm	N/R
Washington et al., 2008 [13]	Fever, dysuria, septicemia and shock	Procidentia	3 years	CT abdomen + Pelvis	Vaginal hysterectomy, AR, SSLF	Transvaginal cystolithotomy	Multiple	8.6 cm x 6.8 cm x 4.6 cm	N/R
Dahiya et al., 2007 [14]	Dysuria, difficulty passing urine	Procidentia, cystocele + rectocele	15 years	Pelvic X-ray	Vaginal hysterectomy, APR, perineorrhaphy	Transvaginal cystolithotomy	15	N/R	N/R
Megadhana et al., 2006 [15]	Dysuria	Incarcerated procidentia	9 months	N/R	Vaginal hysterectomy, colpocleisis	Transvaginal cystolithotomy	3	Each 5 cm	N/R
Wai et al., 2003 [16]	SUI, UUI, recurrent UTI	Complete vault prolapse	N/R	N/R	Burch, colpocleisis	Transabdominal cystolithotomy	multiple	2.5 x 1.1 x 0.7 cm	N/R
Dalela & Agarwal, 1999 [17]	Urinary incontinence	Grade 3 uterine prolapse	N/R	Cystoscopy, Pelvic X-ray	N/R	Transabdominal cystolithotomy	5 cm x 3 cm x 1.5 cm	N/R	N/R
Nieder et al., 1998 [18]	Frequency, UUI, dysuria	Cystocele + rectocele grade 4	1 year	Pelvic X-ray	APR, perineorrhaphy	Transabdominal cystolithotomy	N/R	N/R	118 g
Pranikoff et al., 1982 [19]	Cachectic or dehydrated, stone seen blocking external urethral meatus that was effluxed with reduction of the prolapse	Grade 3 uterine prolapse, rectal prolapse	N/R	Pelvic x-ray Excretory urography	No POP corrective surgery, only pessary	Transvaginal cystolithotomy	N/R	N/R	N/R
Gregoir et al., 1976 [20]	He reported 14 cases	Ureteric obstruction with uterine prolapse	N/R	N/R	N/R	N/R	N/R	N/R	N/R
Mahran, 1971 [21]	Urine incontinence, suprapubic pain	Complete procidentia	35 yrs	X-ray Pyelography	Refused surgical treatment for POP	Transvaginal cystolithotomy	9	5 x 8 cm	N/R
Mann et al., 1958 [22]	Passage of calculi	Procidentia, large cystocele and rectocele	5 years	Intravenous pyelogram	Vaginal hysterectomy, BSO, burch, enterocele repair	Transabdominal cystolithotomy	20	3.2 cm x 3 cm x 2.8 cm	260 g
	N/R	Procidentia, large cystocele and rectocele enterocele	23 yrs	IVP	Vaginal hysterectomy, burch, enterocele repair, PR, colpoclesis	Transabdominal cystolithotomy	13	4.4x2.5x2 cm	105 g
Vartan, 1946 [23]	Disturbance in micturition, urinary retention with overflow	Complete uterine prolapse	55 years	N/R	Le-Fort colpocleisis	Transabdominal cystolithotomy with a suprapubic catheter	1	6.7 cm x 4.1 cm x 3.1 cm	49 g
Drew, 1915 [24]	Foul urine, chronic cystitis	Vault prolapse	10 years	N/R	Lefort colpocleisis, colpoperineorrhaphy	Transabdominal cystolithotomy	N/R	N/R	N/R

Discussion

Bladder stones have been a common medical condition, the first reported bladder stone dates back to ancient Egypt [25]. Vesical urolithiasis, better known as bladder stones, comprises minerals such as calcium, oxalate, phosphate, and uric acid. Various factors can contribute to their formation, such as urinary tract infections (UTIs), bladder outlet obstruction, urinary stasis, and metabolic disorders [26,27]. Anatomical differences make bladder stones more common in men, but bladder stones can also affect women, especially in women with risk factors such as urinary tract abnormalities or chronic catheterization [28]. The presentation of vesical calculi includes lower urinary tract symptoms (LUTS) such as urinary frequency, urgency, dysuria, hematuria, and suprapubic pain [26,28]. If untreated, bladder stones can lead to complications such as recurrent UTIs, bladder outlet obstruction, and renal damage [26].

Pelvic organ prolapse is the herniation of the female pelvic organs (bladder, uterus, small or large bowel, or vaginal cuff post hysterectomy) through or beyond the vaginal walls due to weakened pelvic floor support [29]. An observational study conducted between 1990 and 2019 suggested the prevalence of POP globally to be around 40%, and the incidence is expected to increase with age [30]. The pathogenesis of POP is multifactorial, including but not limited to childbirth, trauma, hormonal changes, connective tissue disorders, obesity, chronic coughing, and constipation [31].

Women with POP often present with symptoms of vaginal bulge, pelvic pressure, urinary incontinence, and sexual dysfunction [32]. POP can affect women's quality of life, leading to physical and psychological discomfort and distress. POP can cause limitations in daily activity [33]. POP rarely results in severe morbidity and mortality. On the other hand, if left untreated, particularly in advanced cases, it might lead to bladder outlet obstruction and obstructive uropathy [29]. POP might also lead to a well-recognized complication such as hydronephrosis, which can be defined as a dilation of the kidneys, renal pelvis, and calyces due to impairment of the urine flow [34]. One systematic review estimated the prevalence of hydronephrosis in women with POP from 3.5% to 30.6% [35]. Nonetheless, severe manifestations of advanced POP, such as acute renal failure, uremia, septicemia, or end-stage kidney disease, are rare and only documented in case reports [13,19].

Vesical calculi and POP have been considered separate entities, but recent studies suggest a potential link between the two. Numerous case reports and case series describe a relationship between bladder stones and POP in women, raising a question about a possible causative relationship or shared risk factors. Some researchers hypothesize that the presence of POP may contribute to the development of bladder stones by causing urinary stasis or altering voiding dynamics. Our search also highlighted this association, as most cases had advanced chronic pop. The most prolonged duration of prolapse was 55 years, reported by CK Vartan (1946). On the other hand, two cases had a POP duration of one year [18,10] or less as reported by Megadhana et al. (2006) [15]. This suggests that bladder stone formation in such cases is multifactorial in origin and not only related to urinary stasis with advanced prolapse. Some researchers suggest that bladder stone formation might be attributed to the weakening of the pelvic floor support structures, chronic UTI, and anatomical abnormalities [1,4,5,11,24]. However, the exact nature of this association remains poorly understood, and further investigation is needed to elucidate the underlying mechanisms.

Comparing our case with the literature, from the data we collected, we found that postmenopausal women experience symptoms of vaginal bulge and urinary complaints, similar to our patient. The presence of bladder stones was incidental in most cases, as they were discovered during imaging or intraoperative procedures. This suggests that bladder stones may often go unnoticed or undiagnosed in patients with POP unless specifically investigated. As a result, physicians should have a high index of suspicion for bladder stones, especially in patients with advanced chronic POP, and imaging modality should be considered during preoperative investigations.

American Urology Association and American College of Radiology consider CT scan the gold-standard modality for evaluating patients suspected to have renal calculi. However, other imaging modalities, including Ultrasound (US) and X-rays, can also detect urolithiasis to a lesser extent, as multiple factors, including the composition of the stone, body habitus of the patient, and pregnancy status, play a role in the detection power of each of these modalities [36].

In our case, the renal US was used for preoperative imaging and missed the largest bladder stone. In retrospect, if another imaging modality had been used, the large bladder stone might have been detected preoperatively. This stresses the importance of proper preoperative imaging for precise diagnosis that eventually will play a significant role in counseling the patient and decisions regarding the surgical approach.

It is also important to determine when both POP surgery and vesicle stone removal should be performed. As noted in our literature review, most opted for simultaneous removal of bladder stones and POP surgery. In one case published by (CK Vartan et al., 1942), bladder stones were removed nine months later as the diagnosis was initially missed. Despite having limited data, theoretically, considering an interval between bladder stone removal and POP correction surgery seems to have no significant consequences, this might be an option in centers with no service to treat either POP or bladder stones.

Also, chronic advanced POP and incarcerated procidentia might lead to vaginal infections and ulcers, especially in postmenopausal women. Vaginal tissue in poor condition is more likely to suffer from infection, and the healing process of wounds is more challenging after surgery. As a result, in such cases, it is prudent to delay surgery and utilize medical treatment such as vaginal estrogen cream. The concomitant use of pessaries or vaginal packing will also expedite vaginal tissue healing by reducing friction and improving blood supply. This approach was noted in both our case and the case reported by Yuan et al. (2022). Surgery was planned after vaginal tissue was well estrogenized and ulcers healed with healthy vaginal tissue [37].

We noted variations among reported cases regarding mode management. In our case, the patient underwent a vaginal hysterectomy with sacrospinous fixation, and the bladder stones were successfully removed vaginally through a cystostomy. This approach was similar to the cases reported by Naidu et al. (2011) and Dahiya et al. (2007), who also performed vaginal hysterectomy and transvaginal cystolithotomy. However, other cases employed different surgical techniques. The majority were done transabdominally with open cystolithotomy as reported by the following case reports [5,6,11-13,16-18,22,23]. Only in one study, they reported transurethral cystolithotomy [8].

A question arose regarding the probability of fistula formation after vaginal approach cystolithotomy as that was noted to be a concern of some surgeons in the literature review, leading them to avoid removing bladder stones through the vaginal approach. Upon reviewing available data, only one case of vaginal cystolithotomy patients reported by Pranikoff et al. (1982) developed postoperative vesicovaginal fistula, it was small in size and was successfully managed conservatively with a Foleys catheter for four weeks.

On the other hand, one review stated that the best and most common method of treating bladder stones, in general, is endoscopic transurethral fragmentation of the stone (cystolithotripsy) [28]. As seen in the case published by Herimath et al. (2019), cystolithotripsy could be considered as a treatment option in patients with advanced POP who have been found to have bladder stones preoperatively by imaging, with a following surgery to be planned later on for the management of POP after total removal of bladder stones especially when patients condition or available resources don't permit for concurrent POP surgery. However, in the end, the surgical approach for treating bladder stones and the timing of surgery should be curated according to individual patient characteristics, surgeon preference, and available resources.

It was noted that the number and size of bladder stones varied among cases. Our case showed multiple small stones and a large stone measuring approximately 4 x 4 cm, while other cases reported multiple stones of different sizes. For example, Naidu et al. (2011) removed 30 stones, the largest measuring 2.5 x 3.0 cm, and Mann et al. (1958) removed 20 stones, the largest weighing 260 g. The size and number of stones may influence the surgical technique chosen for their removal.

Furthermore, postoperative care and follow-up varied among the cases concerning the duration of catheterization. In our case, the patient had a Foley catheter in place until a CT cystogram was performed on the 14th day post-op, which showed an intact bladder and led to the removal of the catheter. This approach is similar to the cases reported by Hudson et al. (2014) and Yuan & Ng (2022). CK Vartan et al. (1942) used a suprapubic catheter in his case. Another case reported by Rajamatheswari et al. (2012) used both suprapubic and urethral catheterization and was kept for a total of six weeks prior to its removal. The differences in postoperative care emphasize the lack of standardized guidelines for managing bladder stones in the context of POP.

We would love to highlight that the cases reviewed are individual reports. Similarities and differences observed among these cases shed light on the co-existence of vesical calculi and female POP. However, further studies are needed to improve our understanding of this association's epidemiology, pathophysiology, optimal management, and long-term outcomes.

## Conclusions

In conclusion, our case report highlights the association between vesical calculi and female high-grade POP. Managing bladder stones in the context of POP can be challenging and vary significantly, reflecting the individual patient characteristics and surgeon preferences. The lack of standardized guidelines for managing bladder stones in the presence of POP highlights the need for further research.
